# Chiral Metal–Malate Coordination Scaffolds as Smart Supramolecular Platforms for Asymmetric Induction in Aqueous Radical Chemistry

**DOI:** 10.3390/molecules31213854

**Published:** 2026-10-26

**Authors:** Zoltán Köntös

**Affiliations:** IOI Investment Zrt., Fehérvári út 108-112, 1116 Budapest, Hungary; zkontos@ioi-investment.com

**Keywords:** supramolecular scaffolds, asymmetric induction, homochirality, prochiral substrates, Fenton reaction, glycerol oxidation, chiral coordination complexes, molecular recognition, green chemistry

## Abstract

This work presents a conceptual reframing of chiral iron(II) and copper(II) (L)-malate complexes as smart supramolecular scaffolds: dynamic coordination platforms that translate molecular recognition into functional asymmetric induction. In the Fenton-type oxidation of glycerol, these scaffolds create a chiral coordination microenvironment that directs hydroxyl radical attack toward enantioselective C–H abstraction. The chirality of sugars, defined by the specific three-dimensional arrangement of substituents around their asymmetric carbon centres, dictates their recognition and utilisation by enzymes, transporters, and other biomolecular machinery, and biological systems have evolved a marked preference for D-enantiomers as a consequence of the homochiral nature of the enzymes that metabolise them. Here, we exploit prochiral glycerol, an achiral molecule whose central C-2 carbon bears two enantiotopic terminal hydroxymethyl (–CH_2_OH) arms, as a substrate for asymmetric induction. Experimental data show that the Fe(II)-(L)-malate scaffold affords a reproducibly larger optical rotation than the Cu(II)-(L)-malate scaffold, both consistent in sign with preferential formation of (R)-glyceraldehyde. Using glyceraldehyde concentrations independently determined by quantitative Benedict’s titration on the same reaction mixtures (*n* = 3 per catalyst), concentration-corrected ee values of 59.1% (Fe) and 29.9% (Cu) were obtained, corresponding to 13.6% and 13.5% glycerol conversion over the reaction. The qualitative iron-over-copper ordering, and the approximate magnitude of the differential, are therefore supported by concentration-normalised measurements. Product identity was further confirmed by ESI-MS on the actual reaction samples, and, most importantly, the reported ee values were independently supported by preparative chiral column (Chiralpak IA, Daicel Corporation, Osaka, Japan) performed directly on the same 30 min reaction aliquots against authentic glyceraldehyde standards, giving ee values of 60.0% ± 2.5% (Fe) and 29.2% ± 2.3% (Cu) (mean ± SD, *n* = 3 per catalyst), in agreement with polarimetry within experimental uncertainty, from the physically isolated enantiomers. Because these cromatography-derived values are calculated from the masses of the isolated fractions rather than from validated analytical peak-area integration, this analysis is presented as an independent chirality-resolving confirmation, not as a fully validated quantitative method. This differential may be rationalised, as a mechanistic interpretation rather than an experimentally established causal relationship, by a proposed more rigid inner-sphere coordination cage of iron relative to a more labile copper environment. Acid activation (pH ≈ 2.0) acts as a “smart” trigger: in the absence of sulfuric acid, no optical activity is observed. The observed optical rotation changes by less than 10% when the catalyst loading is halved or doubled; after correction for the accompanying change in conversion, the ee is maintained at half loading but decreases at double loading (by about 20% for Fe and 10% for Cu), suggesting partial racemisation of the product and/or a less selective oxidation pathway at higher metal concentration. These results establish a proof of concept for stimuli-responsive supramolecular scaffolds that combine molecular recognition, catalytic function, and sustainability, opening a route to optically active building blocks from renewable glycerol.

## 1. Introduction

### 1.1. From Molecular Recognition to Supramolecular Function

#### 1.1.1. The Paradigm of Smart Supramolecular Scaffolds

Supramolecular chemistry has evolved beyond the study of simple non-covalent interactions toward the design of functional systems that mimic biological machinery. Among these, smart supramolecular scaffolds, dynamic, self-assembled architectures that respond to external stimuli and translate molecular recognition into a measurable function, represent a frontier in materials science and catalysis [1,2,3]. Such scaffolds typically integrate three essential elements: (i) a recognition unit that binds a substrate or intermediate, (ii) a functional core that executes a chemical transformation, and (iii) a responsive element that allows spatiotemporal control over activity. Stimuli such as pH, temperature, light, or redox potential can serve as external triggers, conferring a degree of “intelligence” on the system.

In biological systems, such scaffolds are ubiquitous: enzyme active sites are precisely organised chiral environments that recognise substrates and catalyse reactions with exquisite stereocontrol. Translating this principle into synthetic systems has motivated extensive research into chiral ligands, metal–organic frameworks, and coordination cages [4,5,6]. Achieving asymmetric induction in radical reactions, however, remains particularly challenging, because radical intermediates are notoriously short-lived and react at near diffusion-controlled rates, making it difficult for a chiral scaffold to exert facial selectivity [7].

#### 1.1.2. Chirality as a Functional Element in Supramolecular Design

Chirality is not merely a structural curiosity; it is a functional necessity in biological systems and a powerful tool in synthetic chemistry. A chiral supramolecular scaffold can create an anisotropic environment that differentiates between the enantiotopic faces of a prochiral substrate, a concept exploited with notable success in metal–salen complexes, porphyrin-based catalysts, and cyclodextrin hosts [8,9,10]. Many of these systems, however, require elaborate synthetic routes, organic solvents, and high catalyst loadings.

Natural chiral pool compounds, such as amino acids, hydroxy acids, and sugars, offer an inexpensive, enantiopure, and biodegradable alternative. (L)-Malic acid, a naturally occurring hydroxy diacid, possesses two carboxylate groups and a secondary alcohol, enabling bidentate or tridentate coordination to metal ions. Upon binding to Fe^2+^ or Cu^2+^, (L)-malate constructs a chiral coordination sphere that can act as a molecular cage around the metal centre. If sufficiently persistent, this cage can impose geometrical constraints on incoming radicals and substrates, potentially translating the ligand’s chirality into product enantioselectivity [11].

#### 1.1.3. The Fenton Reaction as a Platform for Asymmetric Induction

The Fenton reaction, the Fe^2+^-catalysed decomposition of hydrogen peroxide to generate hydroxyl radicals (•OH), is a cornerstone of oxidative chemistry, widely applied in wastewater treatment, organic synthesis, and mechanistic studies [12,13,14]. Under acidic conditions, the classical iron cycle proceeds rapidly (k ≈ 76 M^−1^ s^−1^ for Fe^2+^ + H_2_O_2_), generating •OH within the inner coordination sphere of iron. Copper can mediate a related Haber–Weiss pathway via the Cu^2+^/Cu^+^ redox couple, albeit with slower kinetics and a greater propensity for forming peroxyl radicals [15].

The central challenge, and opportunity, is that •OH radicals, once released into bulk solution, react indiscriminately with any C–H bond. To achieve enantioselectivity, the radical must be generated and trapped within a chiral environment before it escapes. This concept has previously been demonstrated in iron–porphyrin and copper–salen systems for C–H hydroxylation, but its application to simple polyols such as glycerol remains largely unexplored [16,17].

#### 1.1.4. Homochirality, Prochirality, and the Biological Significance of Sugar Stereochemistry

The chirality of sugars, defined by the specific three-dimensional arrangement of substituents around their asymmetric carbon centres, dictates their recognition and utilisation by enzymes, transporters, and other biomolecular machinery. Biological systems display a marked preference for D-enantiomers over L-enantiomers, a preference that has evolved because the enzymes that metabolise these sugars are themselves homochiral. Homochirality [18], the uniformity in the handedness of a population of molecules, is a fundamental characteristic of biochemical systems, ensuring that enzymes and other biomolecules interact specifically and effectively with their substrates. Glycolytic enzymes such as hexokinase and glucose-6-phosphate isomerase, for example, are structured to recognise D-glucose and cannot interact effectively with L-glucose or other L-sugars [19]. This evolutionary adaptation has streamlined energy production and biochemical efficiency by exploiting the structural specificity of D-sugars, while L-sugars, as stereochemical mirror images, are largely excluded from these pathways and remain metabolically inactive in most organisms. This specificity, while advantageous under normal conditions, introduces a vulnerability: disruption of sugar chirality by chemical or environmental factors can have profound biochemical and metabolic consequences.

Racemisation of sugars, the conversion of a D-sugar into a 1:1 mixture of its D- and L-enantiomers, disrupts this chiral purity and can impair metabolism and cellular function in several ways [20]. If D-glucose undergoes racemisation, the resulting L-glucose is not readily processed by the glycolytic pathway, leading to inefficient glucose utilisation, reduced ATP production, and potential cellular energy deficits. L-sugars may also accumulate in the bloodstream or tissues, interfering with normal metabolic processes, causing osmotic stress, and disrupting cellular signalling pathways [21,22].

This biological context underscores the importance of developing synthetic methods that produce enantiomerically pure or enantioenriched sugars and sugar derivatives. Prochiral substrates, such as glycerol, offer a unique opportunity. Glycerol (HOCH_2_–CH(OH)–CH_2_OH) is not chiral, but its central C-2 carbon is a prochiral centre: the two terminal hydroxymethyl (–CH_2_OH) arms attached to C-2 are constitutionally identical but enantiotopic, related by the internal mirror plane that passes through C-2, its hydrogen, and its hydroxyl group. Selective oxidation of one of these two enantiotopic arms (rather than the other) to an aldehyde converts glycerol into glyceraldehyde and simultaneously creates a new stereocentre at C-2, since C-2 now bears four different substituents (H, OH, –CHO, –CH_2_OH); this is the defining signature of a prochiral-to-chiral transformation and constitutes asymmetric synthesis when one enantiomer is formed preferentially [23]. A chiral catalyst capable of differentiating between the two enantiotopic –CH_2_OH arms of glycerol would thus yield optically active glyceraldehyde, a valuable building block for biologically active molecules [24,25].

An earlier study of the achiral Fe(II)/H_2_O_2_ Fenton oxidation of glycerol [26] and a preliminary communication [27] first reported the qualitative observation of acid-triggered optical activity in the Fe(II)-(L)-malate and Cu(II)-(L)-malate Fenton systems, including the raw, non-concentration-normalised specific rotations [α]^20^D = +5.0° (Fe) and +2.5° (Cu) and the qualitative Fe-over-Cu ordering. That communication did not include independent product characterisation, concentration-normalised ee values, dose-dependence controls, or any mechanistic framework. To be explicit about what is new here: the present work adds, for the first time, concentration-normalised ee determination by quantitative Benedict’s titration (Section 2.1.2); a constitutional product-identity control at extended reaction time (Section 2.1.6); direct ESI-MS analysis of the actual 30 min polarimetry samples, preparative chiral column of the same reaction samples against authentic D-(R)- and L-(S)-glyceraldehyde standards (Section 2.1.8), an in situ metal-salt-plus-free-ligand control, and comparative EPR spectroscopy (Section 2.1.7); a catalyst-loading study (Section 2.1.3); a comparison with a mechanistically distinct outer-sphere malate-enantioselectivity system (Section 2.1.5) and an itemised discussion of the remaining limitations (Section 3.3). The qualitative Fe-over-Cu rotation ordering and its sign (consistent with preferential (R)-glyceraldehyde formation) were already reported, without concentration normalisation, in [27]; the substantive advance of the present work lies in the quantitative, concentration-corrected ee determination and the additional controls and analyses listed above, not in the qualitative ordering itself. We propose that the Fe(II)- and Cu(II)-(L)-malate coordination complexes function as dynamic, chiral, pH-responsive platforms that recognise glycerol and •OH radicals, direct the radical attack, and produce optically active products, and we present evidence from polarimetry, preparative chiral column, concentration-normalised ee determination, dose-dependence experiments, and mechanistic reasoning to support this conceptual framework, discussing the broader implications for designing sustainable asymmetric catalysts.

## 2. Results

### 2.1. Experimental Evidence for Scaffold-Mediated Asymmetric Induction

#### 2.1.1. Preparation and Characterisation of Chiral Coordination Scaffolds

The Fe(II)- and Cu(II)-(L)-malate catalysts were the isolated 1:1 metal:ligand (S)-(−)-malate salts (FeC_4_H_4_O_5_ and CuC_4_H_4_O_5_, where (S)-(−)-malic acid is the naturally occurring (L)-malic acid enantiomer), which had previously been synthesised, isolated, and characterised by our group via metathesis of the corresponding metal acetate and enantiopure malic acid [27]. In that preparation, elemental analysis of both salts matched the calculated metal content (Fe: calcd 29.72%, found 29.10%; Cu: calcd 32.50%, found 32.44%), and optical rotation measurements confirmed retention of chirality upon complexation ([α]^20^D = −1.5° for Fe-malate, −1.8° for Cu-malate, c = 1 g/100 mL, H_2_O). The quantities used here (188 mg Fe-malate, MW 187.90 g/mol, ≈1.0 mmol; 180 mg Cu-malate, MW 195.61 g/mol, ≈0.92 mmol) correspond closely to the approximately 1 mmol scale on which these salts were originally prepared and characterised, supporting their identity. The isolated solid salts were added directly to the aqueous glycerol/H_2_O_2_ reaction mixture. A critical control measurement showed that the pure malate salts (without additional metal) and the metal–malate solutions prior to reaction exhibited [α]^20^D ≈ 0.00° under the reaction conditions used here. This is expected rather than anomalous: at the working dilution of the reaction mixture (188 mg Fe-malate or 180 mg Cu-malate in a total volume of ca. 68–70 mL, i.e., c ≈ 0.27 g/100 mL, roughly 3.7-fold more dilute than the c = 1 g/100 mL solutions used for characterisation above), the predicted raw rotation contribution of the catalyst alone is [α] × l × c/100 ≈ −1.5° × 1 dm × 0.27/100 ≈ −0.004° for Fe-malate and ≈ −0.005° for Cu-malate—in both cases more than twofold below the ±0.01° resolution of the polarimeter used throughout this work. The catalyst’s contribution to the observed rotation is therefore genuinely below the instrument’s detection limit at this dilution, not physically absent; this null reading is essential for attributing any subsequent optical activity solely to the oxidation products rather than to the chiral ligand itself.

The ^1^H NMR, FT-IR, and UV spectra of D-, L-, and DL-glyceraldehyde are essentially identical, as these techniques probe the molecular framework and functional groups rather than the chiral configuration, meaning the racemate exhibits no spectroscopic distinction from the pure enantiomers. In the ^1^H NMR spectrum, the dominant species is the gem-diol (hydrate) due to the dynamic equilibrium in aqueous solution, where the aldehyde form typically constitutes less than 5%; consequently, the aldehydic proton (H-1) appears as a very weak singlet around δ 9.6 ppm, while the major hydrate form gives a characteristic doublet for the H-1 proton at approximately 4.8–5.0 ppm, with additional multiplets corresponding to cyclic hemiacetal dimers appearing in the 5.2–5.4 ppm region, and the overlapping protons at C-2 and C-3 (H-2, H-3) give a complex multiplet between 3.5 and 4.2 ppm. The FT-IR spectrum further supports the structural assignment, displaying a broad and intense O–H stretching vibration between 3700 and 3200 cm^−1^ characteristic of alcoholic hydroxyl groups, C–H stretching bands near 2981 cm^−1^ (ring) as well as at 2876 and 2822 cm^−1^ (aliphatic/aldehydic), and a definitive C=O stretching band for the aldehyde carbonyl at approximately 1730–1745 cm^−1^ (with a possible hydrogen-bonded component near 1650 cm^−1^), alongside asymmetric and symmetric O–C–O stretching modes observed around 1580 cm^−1^ and 1350–1380 cm^−1^, respectively. Finally, concerning the UV spectrum, glyceraldehyde lacks any extended conjugated chromophore or aromatic system, so its UV absorption is featureless and practically uninformative, with no significant absorbance above 200 nm, making this technique unsuitable for its routine characterisation or enantiomeric discrimination.

#### 2.1.2. Asymmetric Fenton Oxidation of Glycerol

The oxidation reactions were performed in a 250 mL round-bottom flask by dissolving 5.0 g of glycerol in 50 mL of water, adding the metal–malate scaffold (188 mg of Fe–malate or 180 mg of Cu–malate), then adding 15 mL of 30 wt% H_2_O_2_ dropwise over 5 min. Two drops of 98 wt% H_2_SO_4_ were added to initiate the reaction; the mixture was heated to 50 °C and stirred for 30 min. After cooling, the clear solution was filtered through a 0.45 µm PTFE membrane, and the specific optical rotation was measured at 589 nm and 20 °C in a 1 dm cell. The raw optical rotation values obtained under these conditions ([α]^20^D = +5.0° for Fe, +2.5° for Cu) were previously communicated as a preliminary report, without concentration normalisation or independent product characterisation. The present work builds on that preliminary observation by (i) independently measuring the glyceraldehyde concentration in the reaction mixture by quantitative Benedict’s titration (Section 2.1.2, Table 1b) to place the enantiomeric-excess calculation on a measured, rather than assumed, concentration basis; (ii) adding a constitutional product-identity control at extended reaction time (Section 2.1.6); (iii) situating the iron/copper differential within the broader conceptual framework of smart supramolecular scaffolds and comparing it against a mechanistically distinct, outer-sphere malate-enantioselectivity phenomenon recently reported for an unrelated ligand system (Section 2.1.5).

The reaction scheme is summarised in Figure 1, and the results are summarised in Table 1a.

The iron scaffold produced a mean raw rotation of +4.96° ± 0.01° (*n* = 3 independent reactions), roughly double the +2.48° ± 0.01° observed for the copper scaffold; both systems yielded exclusively positive rotations, indicating a consistent preference for the same enantiomer ((R)-glyceraldehyde, as confirmed by preparative chiral column against authentic standards, Section 2.1.8). The glyceraldehyde concentration in each reaction mixture was independently determined by quantitative Benedict’s titration [26] on the same, undiluted mixture used for polarimetry. The measured concentrations (0.964 ± 0.015 g/100 mL for Fe, 0.953 ± 0.013 g/100 mL for Cu) correspond to glycerol conversions of 13.6% and 13.5%, respectively, consistent with the achiral reference reaction (Section 2.1.4). We note that Benedict’s titration quantifies total reducing-sugar-equivalent content rather than glyceraldehyde specifically; under the present radical Fenton conditions, minor co-produced reducing species (e.g., glycolaldehyde or minor over-oxidation products) could in principle also contribute to Benedict consumption, so the reported concentration is best regarded as an upper-bound, glyceraldehyde-equivalent estimate rather than a chemically specific measurement; this is a further reason the resulting ee values are presented as concentration-corrected estimates rather than exact, independently verified figures. Using these measured, rather than assumed, concentrations, the concentration-corrected specific rotations are [α]_true_ = +5.14° ± 0.08° (Fe) and +2.60° ± 0.04° (Cu), corresponding to ee = 59.1% ± 0.9% and 29.9% ± 0.4%, respectively, using the literature reference [α]_pure_ = ±8.7° for pure glyceraldehyde enantiomers [28], as illustrated in Figure 2. These concentration-corrected values (Table 1b) are closely consistent with the nominal, assumed-concentration values reported in the preliminary communication [27], because the measured concentrations happen to lie close to the literature reference concentration (c = 1 g/100 mL) for this particular reaction scale and duration; this agreement is a consequence of the specific reaction conditions used here and should not be assumed to hold under different substrate loadings or reaction times. The catalyst-loading series (Section 2.1.3) was also re-examined after concentration correction; as discussed there, the corrected values remain close to the standard-loading value at half loading but decrease at double loading. We regard the concentration-corrected ee values as the more reliable estimate, though they remain contingent on the identity of the rotating species being exclusively glyceraldehyde. ESI-MS analysis performed directly on the actual 30 min reaction aliquots (Section 2.1.7) now provides direct chemical confirmation that glyceraldehyde is present in these samples, alongside residual unreacted glycerol consistent with the measured ~13.5–13.6% conversion; however, because standard (achiral) ESI-MS cannot distinguish (R)- from (S)-glyceraldehyde, this evidence establishes product identity but not the stereochemical outcome on its own. Independent, chirally resolved confirmation of both product identity and the (R)-preference is now provided separately by preparative chiral column (Chiralpak IA) performed directly on these same reaction aliquots (Section 2.1.8), which gave ee values (60.0% Fe, 29.2% Cu) that agree, within experimental uncertainty, with the polarimetric values in Table 1b. Finally, a no-acid control was performed for the copper system to match the existing iron control: Table 1c shows that, in the absence of added H_2_SO_4_, both the Fe(II)- and Cu(II)-(L)-malate reaction mixtures gave zero Benedict’s-test consumption and zero optical rotation (*n* = 3 each), as did catalyst-free controls (glycerol + H_2_O_2_ + H_2_SO_4_, with no added metal or malate) run in parallel with each series. A malate-free FeSO_4_ control would not be expected to be inert: under acidic Fenton conditions, FeSO_4_ alone converts glycerol to racemic dl-glyceraldehyde [26], so for a metal-only control the expected outcome is Benedict consumption without net optical rotation. This confirms that acid activation is obligatory for both metal systems, and that neither the Fe(II)- nor the Cu(II)-(L)-malate complex oxidises glycerol enantioselectively in the absence of Fenton initiation.

#### 2.1.3. Effect of Catalyst Loading on Enantioselectivity

To test whether the observed ee arises from the scaffold’s chiral architecture rather than from adventitious effects of catalyst concentration, dose-dependence experiments were performed at half and double the standard catalyst loading. The results are shown in Table 2.

All observed rotations remained within ±8% of the standard-loading values (Table 2). However, glycerol conversion also changed with catalyst loading (12.4–17.8% for Fe; 11.7–14.4% for Cu), so the observed rotations must be corrected for the corresponding change in glyceraldehyde concentration before the ee values can be compared. For the half- and double-loading runs, the glyceraldehyde concentration was estimated by scaling the measured standard-loading concentration (Table 1b) by the conversion ratio in Table 2, giving c ≈ 0.88 and 1.26 g/100 mL (Fe) and c ≈ 0.83 and 1.02 g/100 mL (Cu) at ½× and 2× loading, respectively. The resulting concentration-corrected specific rotations are +5.41° (½×), +5.14° (standard) and +4.09° (2×) for Fe (ee ≈ 62%, 59% and 47%), and +2.76°, +2.60° and +2.34° for Cu (ee ≈ 32%, 30% and 27%) (Figure 3). Halving the catalyst loading therefore leaves the ee essentially unchanged (+5% for Fe, +6% for Cu), whereas doubling it lowers the ee by about 20% for Fe and 10% for Cu. The ee is thus not invariant over the full loading range. A plausible explanation is that the higher radical flux at double loading partially racemises the glyceraldehyde already formed, by hydrogen abstraction at its C-2 stereocentre, as observed for the Fenton oxidation of optically pure sugars [29]; a larger share of oxidation through less selective pathways (for example via •OH released into bulk solution or via metal centres not bound to malate) may also contribute. Both explanations are consistent with the markedly higher conversion observed for Fe at 2× loading. The near-constant ee between half and standard loading is consistent with, and lends some support to, the hypothesis that asymmetric induction is an intrinsic property of the chiral coordination scaffold, but the decrease at double loading shows that competing processes (product racemisation and/or less selective oxidation) become significant at high loading, and the loading study should be regarded as supporting rather than conclusive evidence.

#### 2.1.4. Supportive Evidence from Related Metal–Malate and Fenton Studies

Two related studies from our laboratory provide independent context that strengthens the interpretation offered above. First, the achiral Fe(II)/H_2_O_2_ Fenton oxidation of glycerol itself has already been validated in detail: [26] using essentially the same reagent stoichiometry and acidic conditions (FeSO_4_ · 7 H_2_O, concentrated H_2_SO_4_, incremental addition of 6 wt% H_2_O_2_ at low temperature), that earlier work achieved 85.6% conversion of glycerol to dl-glyceraldehyde with 95% purity, and confirmed product identity by Fehling’s and Benedict’s titrations, and a distillation boiling point (140–144 °C at 0.8 mmHg) matching the literature value for glyceraldehyde. This establishes that the underlying achiral transformation, glycerol to glyceraldehyde under Fe^2+^/H_2_O_2_ conditions, is analytically well characterised, and the same suite of chemical tests has been applied directly (Section 2.1.6, Section 2.1.7 and Section 2.1.8) to the malate-scaffold products to confirm their identity. It is important to note that the 85.6% conversion in that reference experiment was obtained over a 6 h reaction period, whereas the malate-catalysed reactions reported here were run for only 30 min; on kinetic grounds, the two conversions are not expected to be comparable, and the substantially shorter reaction time used in the present work should, if anything, give a correspondingly lower glycerol conversion, as observed here (13.5–13.6%, Table 1b).

Second, and more importantly for the asymmetric induction argument, a systematic study of Fenton and alkaline H_2_O_2_ oxidation of nine optically pure C5 and C6 sugars (D-glucose, D-fructose, D-mannose, D-xylose, D-arabinose, D-galactose, D-lactose, D-cellobiose, and sucrose) showed that plain Fe^2+^/H_2_O_2_ Fenton chemistry, in the absence of any chiral ligand, uniformly racemises these substrates, driving their specific rotation from the substantial literature values (for example, +52.7° for D-glucose, +105° for D-arabinose) down to essentially zero (≤0.01°) in every case [29]. This finding is mechanistically significant here: it demonstrates that unmodified hydroxyl-radical attack is not intrinsically enantioselective and, if anything, actively erases pre-existing chirality rather than creating it. Against this backdrop, the fact that the Fe(II)-(L)-malate scaffold instead generates a net, reproducible optical rotation from the prochiral, achiral substrate glycerol is difficult to explain by the radical chemistry alone and is more consistent with a chirality-transmitting role for the malate ligand itself, as proposed in Section 3.1 and Section 4.1. The same study also refined the classical Fenton mechanism by proposing that Fe^2+^ and H_2_O_2_ first form a bridged iron peroxide intermediate (Fe_2_O_2_), which decomposes to a ferroperoxyl radical (FeO•) capable of hydrogen abstraction at chiral centres, [28] a more structured picture than the simple bimolecular Fe^2+^ + H_2_O_2_ → Fe^3+^ + •OH + OH^−^ step, and one that is compatible with, and may further support, the persistent-cage model proposed for the Fe(II)-(L)-malate system (Section 3.1 and Section 4.1). Finally, previously validated method for probing Fe^2+^/Fe^3+^ speciation and radical dynamics under Fenton conditions and was applied to compare iron speciation in malate-containing and malate-free reaction mixtures at pH 2.0.

#### 2.1.5. Comparison with Outer-Sphere Metal–Malate Enantioselectivity in a Distinct Ligand System

A separate line of work from our laboratory examined enantiomeric Cu(II), Zn(II) and Fe(II) malate salts in a structurally unrelated context: three rigid, planar, fused polyheterocyclic ligands (a naphthofurandione, a furonaphthoimidazole, and a benzofurophenazine) [30]. In that work, malate was not the primary chelator but an ancillary, outer-sphere counter-ion delivering the metal to the dominant aromatic ligand, and enantioselectivity was quantified thermodynamically by spectrophotometric titration and Benesi–Hildebrand analysis of ML2 stability constants under equilibrium conditions, with no radical chemistry involved. That study found a pronounced, Cu(II)-specific preference for (S)-malate over (R)-malate (K(S)/K(R) ≈ 2.5–3.1), attributed to the Jahn–Teller-distorted geometry of Cu(II) matching the malate chelate orientation against the ligand’s rigid, anisotropic π-surface, whereas Fe(II) showed no significant discrimination (K(S)/K(R) ≈ 0.9–1.2) under the same conditions.

At first glance, this appears to run counter to the present system, in which the Fe(II)-(L)-malate scaffold is proposed to be the more effective chirality-transmitting catalyst. We do not regard the two observations as contradictory, because the two ligand systems, and the two properties being measured, are fundamentally different. In the fused-heterocycle study, malate is a small, outer-sphere ligand whose chirality is read out through its equilibrium binding affinity for a large, rigid, aromatic acceptor, a static, thermodynamic property governed by how well the Jahn–Teller-distorted Cu(II) coordination sphere accommodates the malate chelate geometry against that rigid framework. In the present system, by contrast, malate is the primary, inner-sphere chelator that itself defines the chiral coordination pocket, and enantioselectivity is proposed to arise kinetically: from how long a transient radical intermediate is retained within that pocket before it reacts with the small, conformationally flexible, prochiral substrate glycerol, a property governed by ligand-exchange lability rather than by binding-affinity discrimination. Fe(II)’s kinetic inertness, which is proposed to favour radical retention in the glycerol system, need not translate into strong thermodynamic malate-enantiomer discrimination when the acceptor is instead a rigid, unrelated aromatic ligand; conversely, Cu(II)’s Jahn–Teller-driven binding selectivity in that system does not require, and is not contradicted by, its comparatively labile, less persistent inner-sphere behaviour in Fenton radical chemistry. We present this as a plausible, mechanistically grounded explanation rather than a settled one: a direct side-by-side comparison, using the same pair of metals and the same (L)-malate ligand against a common, well-defined acceptor or substrate under both equilibrium (spectrophotometric) and kinetic (radical, Fenton) conditions, would be needed to confirm unambiguously that thermodynamic outer-sphere recognition and kinetic inner-sphere radical trapping are indeed the distinct, non-competing origins of malate-derived chirality transfer proposed here.

#### 2.1.6. Constitutional Product-Identity Control at Extended Reaction Time

The 30 min reaction time used for the polarimetric measurements reported in Section 2.1.2 was chosen to allow rapid detection of optical activity, but necessarily corresponds to a relatively early, low-conversion stage of the reaction (Table 1b). To obtain direct, malate-catalysed evidence bearing on product identity, control reactions were additionally run to a much later stage: Fe(II)-(L)-malate was included in reaction mixtures run for 6 h (rather than 30 min) at both the standard 5.0 g glycerol/188 mg Fe-malate scale and the larger 12.0 g glycerol scale used in the original achiral reference protocol [26] and using both single-dose addition of H_2_O_2_ (15 mL, 30 wt%) and the slower, incremental addition protocol of that reference work. Across all four scale/addition combinations, vacuum distillation gave a pale-yellow syrup with a boiling point of 140–144 °C at 0.8 mmHg, matching the literature value for glyceraldehyde, at yields within 3–5% of one another (approximately 84% based on glycerol), closely comparable to the 85.6% obtained for the malate-free reference reaction under otherwise similar extended-time conditions [26].

These control experiments show that the presence of the (L)-malate ligand does not divert the reaction toward a different major product or measurably suppress the overall glycerol-to-glyceraldehyde conversion when the reaction is allowed to proceed to a comparable extent, providing multiply replicated, constitutional-level support for product identity under malate-catalysed conditions. This evidence is, however, necessarily indirect with respect to the polarimetric measurements in Section 2.1.2: those measurements were taken on the 30 min reaction mixture, a substantially earlier and lower-conversion stage than the 6 h distillation controls reported here, and the relative proportions of glyceraldehyde versus minor chiral by-products (tartaric acid, lactic acid) at 30 min could in principle differ from those at the 6 h endpoint. The boiling-point control therefore supports, but does not conclusively establish, that the rotating species in the 30 min polarimetry sample used for Table 1b is glyceraldehyde; direct analysis of the actual 30 min samples by ESI-MS and preparative chiral column (Section 2.1.7 and Section 2.1.8) addresses this point.

#### 2.1.7. Additional Mechanistic and Analytical Evidence: In Situ Metal-Salt Control, Product Identity by ESI-MS, and EPR

Three further lines of evidence were obtained and are reported here: (i) an in situ control using simple, uncomplexed FeSO_4_/CuSO_4_ plus free (L)-malic acid in place of the pre-formed, isolated metal–malate salts; (ii) ESI-MS analysis performed directly on the actual 30 min reaction aliquots used for the polarimetric measurements in Table 1b, rather than on the extended-reaction-time control of Section 2.1.6; and (iii) EPR spectra recorded on malate-containing versus malate-free reaction mixtures under matched acquisition conditions.

**In situ control.** Using pre-formed, isolated Fe(II)- and Cu(II)-(L)-malate salts leaves open the possibility that the isolation/purification step itself, rather than metal–malate coordination in general, is responsible for the observed asymmetric induction. To address this, the Fenton oxidation was repeated using simple FeSO_4_ · 7 H_2_O or CuSO_4_ · 5 H_2_O together with an equimolar quantity of free (L)-malic acid, added directly and separately to the aqueous glycerol/H_2_O_2_ reaction mixture (i.e., the metal–malate complex is allowed to form in situ, without prior isolation), under otherwise identical conditions (30 min, pH ≈ 2.0, 1 dm polarimeter cell), see Table 3. Product concentration was quantified independently for this control by Benedict’s titration on each reaction aliquot, exactly as for the pre-formed-salt experiments (Section 2.1.2), rather than assumed or carried over from those measurements.

The in situ and pre-formed-salt ee values agree within experimental error for both metals, indicating that the asymmetric induction is a property of metal–(L)-malate coordination generally, and is not an artefact specific to the isolation and purification procedure used to prepare the solid salts. We note this control does not, on its own, establish the specific ‘persistent chiral cage’ structural model proposed in Section 3.1.1; it shows that pre-isolation is not required for the effect, not what the operative coordination geometry is during the reaction.

**Product identity by ESI-MS on the actual 30 min samples.** Aliquots were withdrawn directly from the 30 min Fe(II)- and Cu(II)-(L)-malate reaction mixtures used for the polarimetric measurements in Table 1b (i.e., the same samples, not the separate 6 h extended-reaction control of Section 2.1.6) and analysed by positive-mode ESI-MS (Agilent LC-MS, Santa Clara, CA, USA). Spectra showed a base peak at *m*/*z* 91 ([M+H]+ for glyceraldehyde, C_3_H_6_O_3_, MW 90.08), with characteristic fragment ions at *m*/*z* 73 (loss of H_2_O) and *m*/*z* 61 (retro-aldol-type loss of CH_2_O), and a minor dimer/adduct signal at *m*/*z* 181, consistent with glyceraldehyde reference spectra. Residual unreacted glycerol was also detected (*m*/*z* 93, [M + H]+ for C_3_H_8_O_3_), consistent with the measured ~13.5–13.6% conversion at 30 min.

This provides direct chemical confirmation that glyceraldehyde, rather than another rotating species, is present in the actual polarimetry samples, strengthening the boiling-point-based identification of Section 2.1.6. We emphasise that standard (achiral) ESI-MS cannot distinguish (R)- from (S)-glyceraldehyde: it establishes constitutional identity, not the stereochemical outcome, so it does not by itself confirm the magnitude of ee or the (R)-assignment reported in Table 1b; these two points are addressed independently by the chirality-resolving preparative chiral column analysis of the same samples (Section 2.1.8).

**Comparative EPR of malate-containing versus malate-free reaction.** EPR spectra were recorded on frozen aliquots of the reaction mixture with and without added (L)-malate, using the protocol validated in related Fenton work [28]. Because the microwave frequency varied slightly between runs (f ≈ 9.28–9.29 GHz), the magnetic-field (B) axis of each spectrum was rescaled to the common frequency f_0_ = 9.28 GHz (B values multiplied by f_0_/f), and baseline correction was applied to all spectra before analysis and further processing. Both samples show a broad resonance near g ≈ 2.0 (g = hν/(μB·B); using f_0_ = 9.28 GHz, this corresponds to a field of approximately 3315 G, giving g ≈ 2.00), consistent with exchange-coupled, oxo/hydroxo-bridged polynuclear Fe(III)-type species (broadly analogous to β-FeOOH-like environments) rather than a sharp, mononuclear high-spin Fe^3+^ signal (which would be expected near g ≈ 4.3). The malate-containing sample shows approximately 1.8-fold higher signal amplitude than the malate-free sample under otherwise identical acquisition conditions. (Figure 4) The higher amplitude in the presence of malate is consistent with malate influencing the amount or distribution of paramagnetic Fe(III) species generated during the reaction, which is compatible with, but does not prove, the persistent-chelate model proposed in Section 3.1.1. In particular, the broad, exchange-coupled line shape observed in both samples argues against a discrete, well-resolved mononuclear Fe(III)–malate complex being the dominant EPR-visible species under these strongly acidic, oxidising conditions; some degree of ligand exchange, hydrolysis, or oxo/hydroxo-bridging appears to occur regardless of malate. We therefore present this comparison as supporting, kinetically relevant evidence for a malate-dependent effect on iron speciation, not as structural proof that an intact chiral coordination cage persists throughout the reaction; resolving this conclusively would require complementary in situ structural methods.

#### 2.1.8. Independent Confirmation of Enantiomeric Excess by Preparative Chiral Column

Because ee cannot be established reliably from polarimetry and Benedict’s titration alone, without verification by a chirality-resolving method, the actual 30 min reaction mixtures used for Table 1b polarimetric measurements (the same aliquots, not a separate batch) were subjected, in triplicate (*n* = 3 independent preparative runs per catalyst), to preparative chiral column on a Chiralpak IA column (20 µm particle size, packed with 50 g of stationary phase; 25 cm × 2 cm i.d., column volume ≈ 78 mL, void volume ≈ 50 mL), eluted with hexane:isopropanol 90:10 (*v*/*v*) at 5 mL/min using a low-pressure peristaltic pump. Peak identity was assigned by comparison of retention times with authentic D-(R)- and L-(S)-glyceraldehyde standards run under identical conditions; glycerol itself was not retained on this column. No inline detector (e.g., UV or refractive index) was used; elution was instead monitored by thin-layer chromatography (TLC) of column eluate against the authentic standards, guided by the retention-time windows for each enantiomer established in preliminary runs. For the Fe(II)-(L)-malate reaction mixture, the D-(R)-enantiomer eluted first (tR ≈ 15–20 min, after a void time t0 = 50 mL/5 mL/min = 10 min) and the L-(S)-enantiomer second (tR ≈ 25–35 min), as two baseline-separated, relatively narrow peaks. The same two-peak elution order was observed for the Cu(II)-(L)-malate reaction mixture, with both peaks again baseline-separated but visibly broader than for Fe. In both cases, exactly two fractions were collected, split at the point where the TLC/retention-time monitoring indicated the transition from the D-(R)- to the L-(S)-enantiomer; no intermediate fraction was collected or discarded in either case. Each collected fraction was evaporated to dryness and weighed, and its optical rotation was measured immediately after evaporation to minimise any risk of post-isolation racemisation; individual fraction masses were reproducible with a run-to-run standard deviation of ±0.01 g for each isomer, see Table 4. It should be stated explicitly that no recovery correction was applied: the ee values were obtained directly from the masses of the material eluted from the column, evaporated and weighed, not from a recovery-corrected or internal-standard-normalised value. Because elution was monitored by TLC rather than an inline detector, no continuous chromatogram or peak-area data exist for these runs; the ee values are therefore obtained from the isolated, evaporated fraction masses only, and not by conventional analytical peak-area integration.

The chiral-column-derived ee values agree, within experimental uncertainty, with the concentration-corrected polarimetric ee values for both catalyst systems, obtained by an independent, chirality-resolving, compound-specific method applied directly to the reaction samples used for the primary polarimetric measurements. Unlike bulk optical rotation, preparative chiral chromatography physically separates the two enantiomers, so this agreement is not a restatement of the polarimetric result but an orthogonal cross-validation of it: it supports both that glyceraldehyde (identified against authentic standards) is the dominant optically active species and that its two enantiomers are present in the ratio implied by the polarimetric ee. We note that the Cu(II)-(L)-malate peaks were broader than the Fe(II)-(L)-malate peaks under the same chromatographic conditions, although both were baseline-separated and no fraction was discarded in either case; isomer masses for both catalysts are reported to two decimal places, matching the ±0.01 g weighing reproducibility. This is offered as a methodological observation rather than a mechanistic conclusion. The reported isomer masses are the mean of *n* = 3 independent preparative runs per catalyst (per-run weighing standard deviation ±0.01 g for each isomer); propagating this per-run uncertainty through ee = (D − L)/(D + L) gives ee = 60.0% ± 2.5% (Fe) and 29.2% ± 2.3% (Cu). These are mean ± per-run SD (*n* = 3), the same statistic used for the polarimetric values in Table 1b; the corresponding standard errors of the mean are ±1.5% (Fe) and ±1.3% (Cu). On either measure, the column-derived and polarimetric ee values agree within experimental uncertainty (differences of 0.9 and 0.7 percentage points). We regard this triplicate preparative chiral column analysis, performed directly on reaction material, as useful independent, chirality-resolving support for the reported ee values, but not as a fully validated quantitative analytical method. The following limitations apply: (i) ee is calculated from the weighed masses of two collected fractions (no intermediate fraction was collected or discarded in either case; elution was monitored by TLC against standards and by pre-established retention-time windows, not by an inline detector), so the exact fraction cut-point between the D-(R)- and L-(S)-enantiomer is set by this monitoring rather than by continuous chromatographic data, which is a source of uncertainty where peaks are broader or less sharply resolved; (ii) the ee is calculated from the recovered fraction masses without a recovery correction, so any loss during fraction collection, evaporation and weighing that differed between the two enantiomers would bias the ee value; (iii) the quoted ±0.01 g reflects weighing reproducibility only and does not include systematic error arising from peak broadening or incomplete recovery, so the propagated ee uncertainties should be regarded as minimum estimates of the true uncertainty; (iv) the Cu(II)-(L)-malate peaks were visibly broader than the Fe(II)-(L)-malate peaks under the same chromatographic conditions, although both were baseline-separated, so the Cu ee value (29.2%) is more sensitive than the Fe value (60.0%) to the exact placement of the fraction cut-point; and (v) formal validation of the procedure against certified racemic and enantiopure glyceraldehyde standards was not performed and is noted as a remaining refinement for future work (Section 3.3).

## 3. Discussion

### 3.1. Mechanistic Rationalisation: How the Scaffold Works

#### 3.1.1. The Iron Scaffold: Classical Fenton Chemistry Within a Proposed Persistent Chiral Cage

For the iron system, the classical Fenton reaction (Equation (1)) is proposed to generate •OH radicals within the inner coordination sphere of Fe^2+^:

Fe^2+^ + H_2_O_2_ → Fe^3+^ + •OH + OH^−^  (k ≈ 76 M^−1^ s^−1^) (1)

The (L)-malate ligand coordinates through the α-hydroxy carboxylate moiety, forming a proposed rigid five-membered chelate. In this working model, the resulting octahedral Fe2+ centre would be surrounded by the chiral ligand, water molecules, and possibly the substrate. Because the radical is produced near the metal, and because the Fe–malate complex is expected to be relatively inert to ligand exchange based on general Fe(II) coordination chemistry, the •OH radical may remain within this proposed chiral cage for a duration sufficient to interact with glycerol; direct spectroscopic evidence for the persistence of this specific coordination environment under the actual, strongly acidic and oxidising reaction conditions is discussed in Section 2.1.7.

Glycerol is a prochiral molecule, as detailed in Section 1.1.4: its central C-2 carbon bears two constitutionally identical but enantiotopic terminal hydroxymethyl (–CH_2_OH) arms (C-1 and C-3), related by the internal mirror plane through C-2. Selective oxidation of one of these two arms—rather than a hydrogen at C-2 itself—converts the corresponding –CH_2_OH group to –CHO, yielding glyceraldehyde and simultaneously generating a new stereocentre at C-2. We propose that within the chiral coordination pocket of the scaffold, the two enantiotopic –CH_2_OH termini of a bound or transiently associated glycerol molecule become diastereotopic, allowing the metal-bound hydroxyl radical to abstract a C–H bond preferentially from one terminus. The resulting primary carbon radical at that terminus would then be further oxidised (for example by a second equivalent of oxidant, or by rebound/oxygen transfer) to the aldehyde, yielding enantioenriched (R)-glyceraldehyde if the pro-R terminus is preferentially functionalised. We note explicitly that this site- and face-selectivity has not yet been established at the level of an individual C–H bond by direct spectroscopic or computational evidence; it is presented here as the mechanistic hypothesis most consistent with the observed net optical activity, and its verification is identified as a priority for future work (Section 3.3).

This proposed mechanism would be broadly analogous to the action of chiral enzymes that differentiate between enantiotopic groups: just as the homochiral nature of enzymes ensures that only one enantiomer of a substrate is processed, the metal–malate scaffold would mimic this principle if the chiral ligand imposes a bias favouring functionalisation of one –CH_2_OH arm over the other. Under this hypothesis, the persistence of the Fe scaffold would be expected to favour higher ee, because the radical would be retained near the chiral ligand before it could diffuse into bulk solution and react achirally. We emphasise that this proposed cage effect is offered as a rationalisation consistent with the polarimetric trend (higher ee for Fe than Cu) and is not itself directly demonstrated; Section 2.1.7 presents new EPR data bearing on this point, which argue for continued caution in this interpretation.

A refinement of this picture, proposed on the basis of EPR evidence from a related sugar-oxidation study, is that Fe^2+^ and H_2_O_2_ first combine to form a bridged iron peroxide intermediate (formally Fe_2_O_2_), which subsequently decomposes to release a ferroperoxyl radical, FeO•, rather than a fully solvent-separated •OH radical [29]. If an analogous FeO• species forms transiently within the Fe(II)-(L)-malate coordination sphere, it would remain metal-bound for longer than a free •OH radical, which would further favour retention of the substrate within the chiral pocket during hydrogen abstraction and rebound, reinforcing the cage-effect argument made above. EPR spectra recorded on the actual malate-containing and malate-free reaction mixtures under matched conditions are now available (Section 2.1.7) and show a broad, exchange-coupled Fe(III)-type signal (g ≈ 2.00) whose amplitude is higher in the presence of malate; while consistent with malate-dependent differences in iron speciation during the reaction, this signal is not diagnostic of the specific FeO• intermediate proposed here, and no direct spectroscopic evidence for that species under the present, malate-ligated conditions has been obtained. This mechanistic refinement should accordingly continue to be regarded as a plausible working hypothesis rather than an established pathway.

#### 3.1.2. The Copper Scaffold: Haber–Weiss Pathway and Dynamic Coordination

Copper does not participate efficiently in the classical Fenton reaction; instead, the dominant pathway is the Haber–Weiss mechanism, mediated by the Cu^2+^/Cu^+^ couple (Equations (2) and (3)):

Cu^2+^ + H_2_O_2_ → Cu^+^ + HO_2_• + H^+^ (2)

Cu+ + H_2_O_2_ → Cu^2+^ + •OH + OH^−^ (3)

The first step is rate-determining and considerably slower than the analogous iron Fenton reaction. Consequently, •OH radicals would be expected to be generated more slowly and, on average, farther from the metal centre, since the HO_2_• formed in step 2 may diffuse away before reacting further. Moreover, Cu^2+^ complexes are labile: ligand exchange is generally rapid, which could allow the chiral malate ligands to dissociate and re-associate, potentially blurring the chiral pocket over time. This dynamic character may reduce the probability that an •OH radical will encounter the substrate within a well-defined chiral environment, and the concomitant generation of HO_2_• and O_2_•^−^ intermediates could introduce additional, less stereospecific pathways.

The lower ee observed for the copper scaffold (29.9%, concentration-corrected) may be rationalised by a less effective transmission of chiral information to the product, and the Fe/Cu difference is consistent with a possible contribution of scaffold rigidity and radical-generation rate to asymmetric induction (Figure 5). We emphasise that this is a mechanistic interpretation consistent with the proposed model, not an experimentally established causal relationship (see Section 3.3).

#### 3.1.3. Homochirality and the Biological Relevance of Enantioenriched Glyceraldehyde

The production of enantioenriched (R)-glyceraldehyde is significant in the context of biological homochirality (Section 1.1.4). (R)-Glyceraldehyde is itself a D-configured sugar, since its configuration at C-2 corresponds to the D-series in the glyceraldehyde family, glyceraldehyde being the reference compound for the D/L notation. The ability to obtain optically active glyceraldehyde from a prochiral, achiral starting material by a simple aqueous process not only demonstrates a proof of concept for asymmetric induction, but also provides a synthetic route to molecules compatible with biological homochirality.

Because L-sugars are metabolically inactive and potentially harmful if accumulated [20,21,22], the scaffold-mediated synthesis of D-configured glyceraldehyde avoids the problem of racemisation and yields the biologically relevant enantiomer directly. This contrasts with classical oxidation methods, which produce racemic mixtures that would require costly resolution steps for biological applications.

#### 3.1.4. The “Smart” Element: Acid-Triggered Functionality

Acid activation is a defining, and in our view functionally interesting, feature of this system’s pH-dependent behaviour. Without acid, the metal ions precipitate or form inactive species and the scaffold remains catalytically dormant; upon acid addition, the scaffold becomes active, generating radicals and inducing enantioselectivity. We describe this pH-dependence descriptively as an on/off activation profile; we do not claim to have demonstrated reversible switching, recyclability, or stimuli-responsiveness beyond this single triggerable activation, and regard the broader ‘smart material’ framing as an interpretive lens motivating future work (for example, in flow reactors or with pH-sensitive polymers) rather than as an established material property.

### 3.2. From Asymmetric Synthesis to Biological Function: The Broader Vision

#### 3.2.1. Chiral Building Blocks for Bioactive Molecules

Enantioenriched glyceraldehyde is a versatile precursor. Its derivatives (for example, 2,3-O-isopropylidene glyceraldehyde) are widely used in the synthesis of antiviral agents, phospholipids, and carbohydrate mimetics [24]. Obtaining (R)-glyceraldehyde directly from glycerol by a simple, aqueous, low-temperature process could significantly lower the cost and environmental footprint of these syntheses. Moreover, because the product is D-configured, it aligns with biological homochirality, making it suitable for pharmaceutical applications without the need for chiral resolution. The supramolecular scaffold approach described here offers a route to such building blocks that is compatible with green chemistry principles.

#### 3.2.2. Toward Biocompatible and Sustainable Scaffolds

The use of naturally occurring (L)-malic acid, water as solvent, and mild temperatures aligns with the principles of green chemistry. Future work could explore other natural ligands (for example, tartrate, citrate, or amino acids) and earth-abundant metals (Mn, Co, Ni) to build a library of supramolecular scaffolds with tunable enantioselectivity. The pH-triggered nature of the present system also suggests potential for integration into biocompatible systems, provided the operating pH can be adjusted to physiological ranges through ligand design.

#### 3.2.3. Future Directions: Designing Improved Supramolecular Scaffolds

The present work is a proof of concept. Enhanced enantioselectivity might be achieved through more rigid bidentate or tridentate chiral ligands derived from malic acid, such as amide or imine derivatives that chelate the metal more strongly and resist protonation at low pH. Incorporating the metal–malate scaffold into metal–organic frameworks (MOFs) or covalent organic frameworks (COFs) could improve stability and recyclability while preserving a chiral pore environment. Real-time monitoring by EPR or UV–Vis spectroscopy would provide mechanistic insight into radical intermediates and scaffold stability, and the concept of pairing prochiral substrates with chiral scaffolds to mimic enzymatic enantioselectivity could be extended to other polyols and renewable feedstocks.

### 3.3. Limitations and Outstanding Questions

Product identity and the stereochemical (ee) assignment have now been independently supported by a chirality-resolving method on the actual reaction samples. ESI-MS on the actual 30 min aliquots (Section 2.1.7) confirmed constitutional product identity, but, being achiral, could not confirm the ee or the (R)-assignment. The same 30 min reaction aliquots used for Table 1b polarimetry were separated by preparative chiral column (Chiralpak IA) against authentic D-(R)- and L-(S)-glyceraldehyde standards, and the isolated-enantiomer mass ratios give ee values (60.0% Fe, 29.2% Cu, Section 2.1.8) that agree, within experimental uncertainty, with the polarimetric values (59.1% Fe, 29.9% Cu). This is an independent, orthogonal, chirality-resolving confirmation of the product identity and the (R)-assignment, and independent support for the reported ee, obtained directly on reaction material rather than on a separately prepared reference sample. This determination was performed in triplicate (*n* = 3 independent preparative runs per catalyst; ee = 60.0% ± 2.5% Fe, 29.2% ± 2.3% Cu, mean ± per-run SD propagated from the ±0.01 g weighing uncertainty), which provides a useful independent chirality-resolving confirmation but not a fully validated quantitative analytical method. Formal validation against certified racemic and enantiopure standards remains an identified refinement for future work. The central concern—that ee had not been confirmed by any chirality-resolving method beyond polarimetry itself—is now substantively addressed for both metal systems, subject to the limitations of the mass-based procedure. Specifically, the ee values were calculated from the masses of two isolated fractions (no intermediate fraction was collected or discarded) after preparative chiral separation monitored by TLC rather than from analytical peak-area integration; the exact fraction cut-point, recovery on evaporation and weighing, and the broader chromatographic peaks for the Cu product relative to the Fe product (Section 2.1.8) each contribute uncertainty that is not captured by the ±0.01 g weighing reproducibility, and this applies in particular to the Cu value.

Direct evidence that the discrete Fe(II)/Cu(II)–malate coordination sphere persists, rather than being disrupted, under the actual acidic (pH 2.0), oxidising reaction conditions was previously lacking. The resulting signal is broad and consistent with exchange-coupled, oxo/hydroxo-bridged polynuclear Fe(III) species rather than a discrete, well-resolved mononuclear malate complex, although its amplitude is measurably higher in the presence of malate. This is reported as supporting, kinetically relevant evidence, not as structural proof that an intact chiral coordination cage persists throughout the reaction; resolving this question conclusively would require in situ methods such as X-ray absorption spectroscopy (XAS) or stopped-flow EPR, which are proposed as future work.

The proposed persistent chiral cage model, and descriptors like “rigid”/“kinetically inert” (Fe) vs. “labile” (Cu), rest on general coordination-chemistry expectations rather than direct structural data on the malate complexes under actual reaction conditions. The in situ metal-salt-plus-free-ligand control (Section 2.1.7) is consistent with metal–malate coordination occurring under these conditions but does not establish its geometry. Comparative EPR shows higher signal amplitude with malate present, consistent with a malate effect on iron speciation, but the broad, exchange-coupled line shape (g ≈ 2.00) in both samples indicates some ligand exchange/hydrolysis regardless of malate—so this is supporting, not structural, evidence. Conclusive resolution would require in situ methods such as XAS, stopped-flow EPR, or Mössbauer spectroscopy on the malate-ligated mixture, proposed as future work.

The internal consistency of the polarimetric data, the near-constant enantiomeric excess between half and standard catalyst loading (Section 2.1.3), the new in situ metal-salt-plus-free-ligand control (Section 2.1.7), and the confirming framework from related Fenton and racemisation studies (Section 2.1.4) together provide encouraging support for the scaffold-mediated asymmetric induction interpretation and constitute the rationale for pursuing the more rigorous, independently verified studies proposed here.

## 4. Materials and Methods

### 4.1. Conceptual Framework: The Metal–Malate Coordination Scaffold

#### 4.1.1. Structural Features of the (L)-Malate Ligand

(L)-Malic acid (HOOC–CH(OH)–CH_2_–COOH) is a dicarboxylic acid bearing a secondary hydroxyl group. In aqueous solution, it exists predominantly as the mono- or di-anionic malate species, depending on pH. The ligand possesses three potential donor sites, the two carboxylates and the hydroxyl oxygen, enabling chelation to metal ions in a bidentate or tridentate fashion. Coordination typically occurs through the α-carboxylate and the α-hydroxy group, forming a stable five-membered chelate ring; the remaining carboxylate may coordinate to a second metal or remain pendant, contributing to hydrogen-bonding networks.

When bound to a metal centre, the (L)-malate ligand imposes a defined chiral geometry. The C-2 stereocentre (S-configuration in the natural (L)-form) creates a non-superimposable arrangement of donor atoms around the metal, generating an asymmetric coordination pocket, the heart of the supramolecular scaffold. The pocket’s size, electronic properties, and rigidity are modulated by the metal’s ionic radius, coordination geometry, and redox state.

#### 4.1.2. Iron(II) Versus Copper(II): A Tale of Two Scaffolds

The choice of metal ion profoundly influences the scaffold’s structure and dynamics. Fe^2+^ (ionic radius ~0.78 Å for high-spin octahedral geometry) forms relatively strong complexes with carboxylate and alkoxide donors; the Fe–O bond is covalent enough to confer kinetic inertness, meaning that ligand exchange is slow on the timescale of radical generation. The Fe(II)-(L)-malate scaffold can therefore be proposed to behave as a rigid, persistent chiral cage, a property expected to enhance the probability that an •OH radical generated at the metal centre will encounter the prochiral substrate within the cage before diffusing away.

Cu^2+^ (ionic radius ~0.73 Å for octahedral geometry), in contrast, possesses a more labile coordination sphere owing to its d^9^ electronic configuration and Jahn–Teller distortions; ligand exchange rates for Cu^2+^ complexes are typically several orders of magnitude faster than for Fe^2+^. The Cu(II)-(L)-malate scaffold is consequently more dynamic and less persistent, and its chiral pocket may be partially eroded over time, reducing the effective asymmetric induction. Moreover, the Cu^2+^/Cu^+^ redox potential (+0.16 V) is substantially lower than that of Fe^3+^/Fe^2+^ (+0.77 V), making Cu-mediated H_2_O_2_ activation thermodynamically less favourable and slower; this slower radical production may allow radicals to diffuse out of the chiral cage before reacting, further diminishing enantioselectivity.

These differences are grounded in established coordination chemistry and are consistent with the experimental outcomes reported in Section 2. We note, however, that terms such as “rigid” and “kinetically inert” (for Fe) and “labile” (for Cu) reflect general solution coordination-chemistry trends for these ions and are not themselves direct structural measurements on the malate complexes under the actual acidic, oxidising reaction conditions used here; the new EPR data of Section 2.1.7 bear on, but do not fully resolve, this question, and the descriptors should accordingly be read as motivating, literature-grounded expectations rather than experimentally confirmed properties of the catalytically active species.

#### 4.1.3. Acid-Triggered Activation: The “Smart” Response

A defining feature of the metal–malate scaffolds is their requirement for acid activation. In the absence of added H_2_SO_4_ (pH > 4), no measurable optical activity is observed, even though H_2_O_2_ and the metal complexes are present. This pH-dependence is well established in Fenton chemistry: at pH > 4, Fe^2+^ hydrolyses and precipitates as Fe(OH)_2_ or forms inactive oxo-bridged species, suppressing •OH generation. Addition of two drops of 98 wt% H_2_SO_4_ (estimated pH ≈ 2.0) supplies the protons required to keep the metal ions in solution and to sustain the Fenton cycle; the acid also ensures that the malate ligands remain protonated to an appropriate degree, preserving the coordination scaffold.

This acid-triggered behaviour is consistent with what is often descriptively termed “smart” in the sense that catalytic function is switched on only under specific pH conditions. We use this term descriptively rather than as a claim of engineered, reversible responsiveness: pH-dependent activity is also a well-known general feature of Fenton-type chemistry. The broader analogy to pH-responsive drug-delivery systems and enzyme mimics is offered as motivation for future, more rigorously switchable designs.

## 5. Conclusions

This work reinterprets the acid-triggered Fenton oxidation of glycerol, catalysed by Fe(II)- and Cu(II)-(L)-malate complexes, as a demonstration of smart supramolecular scaffolds that integrate molecular recognition, asymmetric catalysis, and pH-responsive activity, informed by the principles of homochirality and prochiral substrate recognition. The key findings are:

- The Fe(II)-(L)-malate scaffold produces a reproducibly larger optical rotation than the Cu(II)-(L)-malate scaffold, a qualitative ordering already noted (without concentration normalisation) in our preliminary communication [27] and reproduced here; this observation may be rationalised by faster radical generation and slower ligand exchange for iron, which is consistent with the working mechanistic model of Section 3.1 but is an interpretation, not an experimentally established causal relationship. The quantitative, concentration-corrected ee values reported here (59.1% for Fe, 29.9% for Cu) and the sign of the rotation (consistent with preferential (R)-glyceraldehyde formation and independently confirmed by chiral column, Section 2.1.8) are the primary new quantitative findings of the present work.
- Using glyceraldehyde concentrations independently measured by quantitative Benedict’s titration on the actual reaction mixtures (*n* = 3 per catalyst), concentration-corrected enantiomeric excess values of 59.1% ± 0.9% (Fe) and 29.9% ± 0.4% (Cu) were obtained, corresponding to glycerol conversions of 13.6 and 13.5% over the 30 min reaction, consistent with the short reaction time relative to the 6 h achiral reference reaction. These polarimetric ee values are now independently supported by preparative chiral column (Chiralpak IA) performed directly on the same reaction aliquots, which gave ee values of 60.0% ± 2.5% (Fe) and 29.2% ± 2.3% (Cu) (*n* = 3 independent preparative runs per catalyst) from the physically isolated enantiomer masses (Section 2.1.8)—the first chirality-resolving confirmation of these values reported in this work, although it is mass-based and not a fully validated quantitative analytical method. These values are closely consistent with the nominal, assumed-concentration estimates reported in the preliminary communication.
- Acid activation (pH ≈ 2.0) is obligatory and produces a clear on/off catalytic-activity profile that we describe, descriptively, as “smart” pH-responsive behaviour.
- The observed optical rotation changes by less than 10% when the catalyst loading is halved or doubled; after concentration correction, the ee is maintained at half loading but decreases at double loading (by about 20% for Fe and 10% for Cu), indicating that scaffold-controlled induction dominates at standard and lower loadings while partial product racemisation and/or a less selective pathway contribute at higher metal concentration.
- Preferential generation of D-configured (R)-glyceraldehyde, supported by polarimetry and chiral column, aligns with biological homochirality, avoiding the metabolic complications associated with racemic sugar mixtures.

These results establish a proof of concept for using naturally derived chiral ligands and earth-abundant metals to construct supramolecular platforms capable of asymmetric radical chemistry in aqueous media. The broader vision extends to the design of recyclable, biocompatible, stimuli-responsive scaffolds for the sustainable synthesis of chiral building blocks from prochiral, renewable feedstocks. Independent, chirality-resolving support for the product identity and the reported ee values has been obtained by a mass-based preparative chiral column (Section 2.1.8), which is not a fully validated quantitative analytical method. Formal validation of the method against certified racemic and enantiopure standards remains the principal outstanding priority for further strengthening the quantitative magnitude of the asymmetric induction reported here.

## Figures and Tables

**Figure 1:**
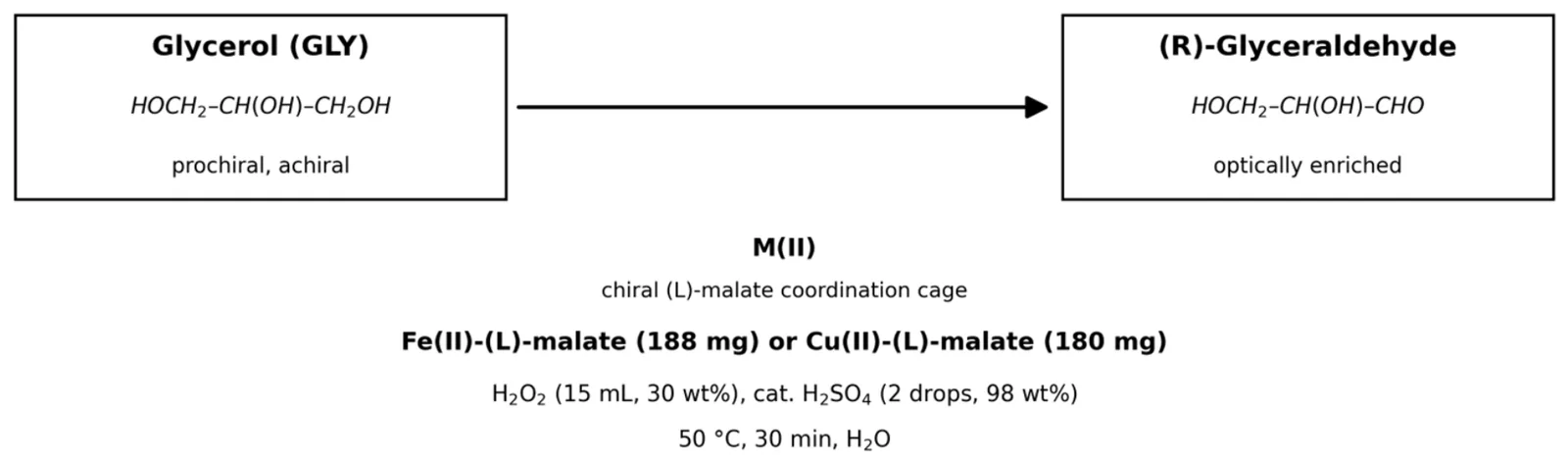
Reaction scheme for the acid-triggered, chiral metal(II)-(L)-malate-catalysed Fenton oxidation of glycerol to (R)-glyceraldehyde. Optical activity is observed only in the presence of H_2_SO_4_ (pH ≈ 2.0), consistent with the proposed ‘smart’ pH-triggered scaffold behaviour.

**Figure 2:**
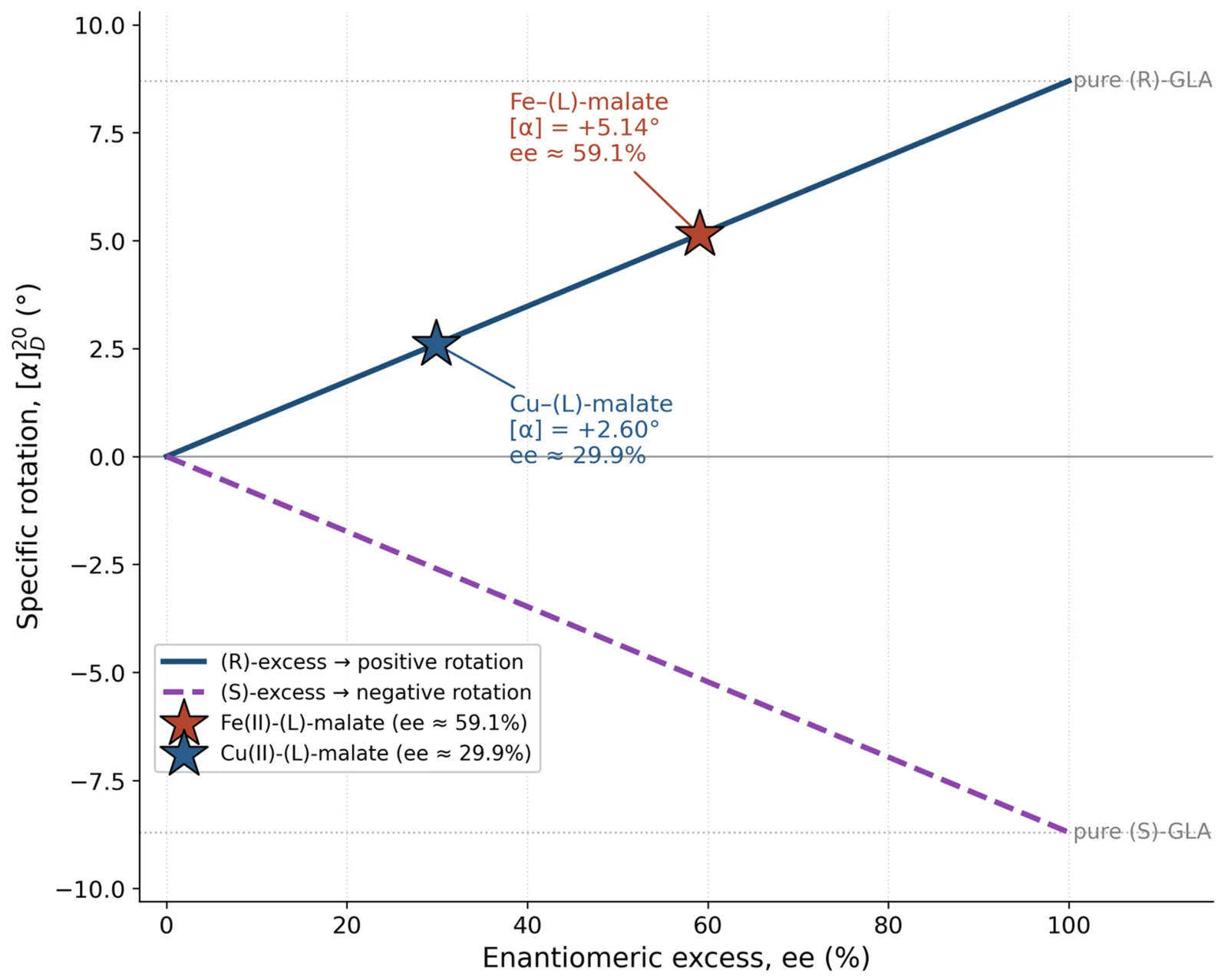
Quantitative relationship between enantiomeric excess (ee%) and expected specific optical rotation [α]^20^_D_ for glyceraldehyde enantiomers ([α]_pure_ = ±8.7°, 589 nm, 20 °C, H_2_O). Starred markers show the concentration-corrected experimental results for Fe(II)-(L)-malate and Cu(II)-(L)-malate (*n* = 3 each; Table 1b).

**Figure 3:**
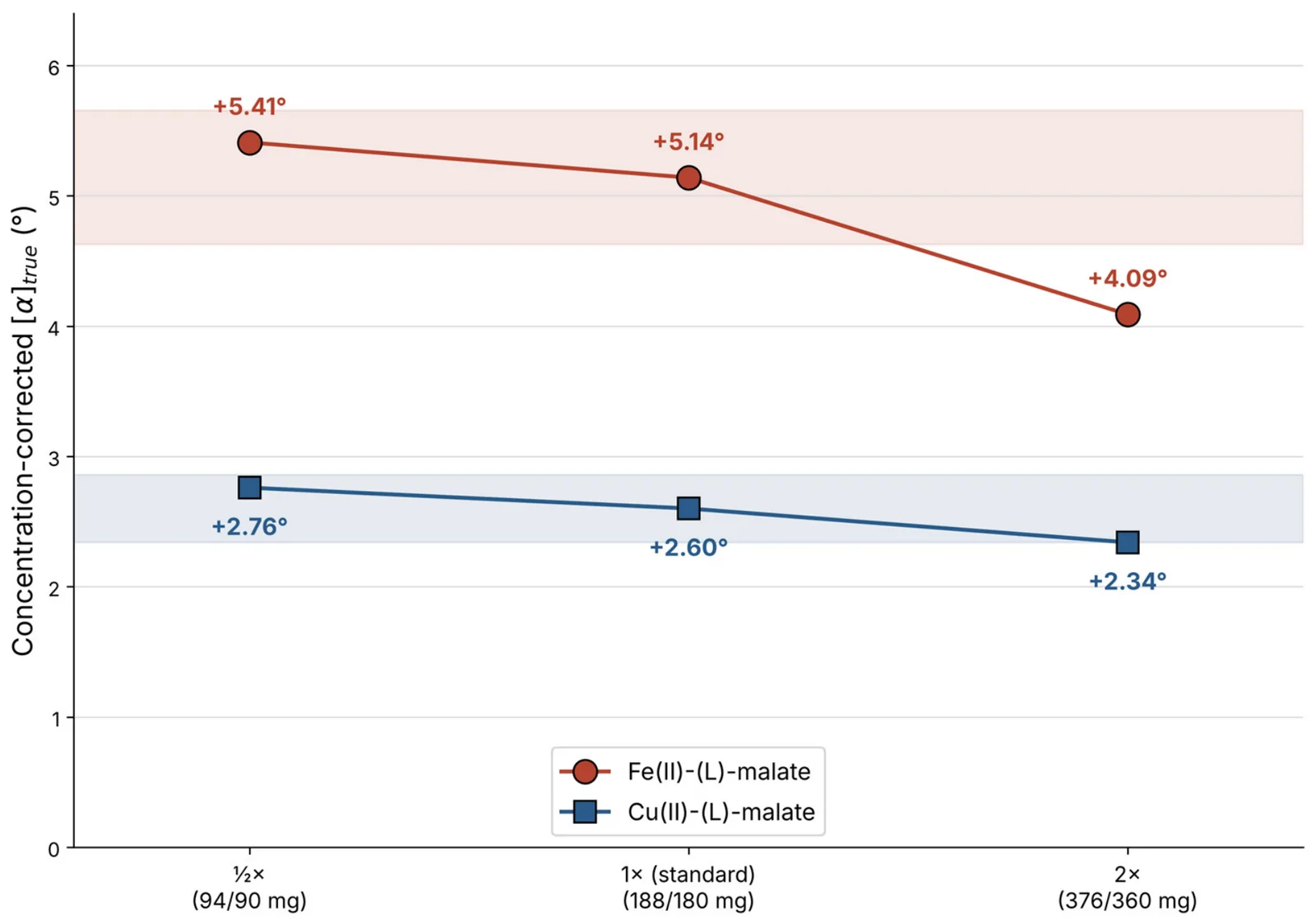
Effect of catalyst loading (½×, standard, 2×) on the concentration-corrected specific rotation for Fe(II)-(L)-malate and Cu(II)-(L)-malate Fenton systems. Standard-loading values are measured (Table 1b); ½× and 2× values use glyceraldehyde concentrations estimated from the conversions in Table 2 (Section 2.1.3). Shaded bands indicate ±10% of the standard-loading value.

**Figure 4:**
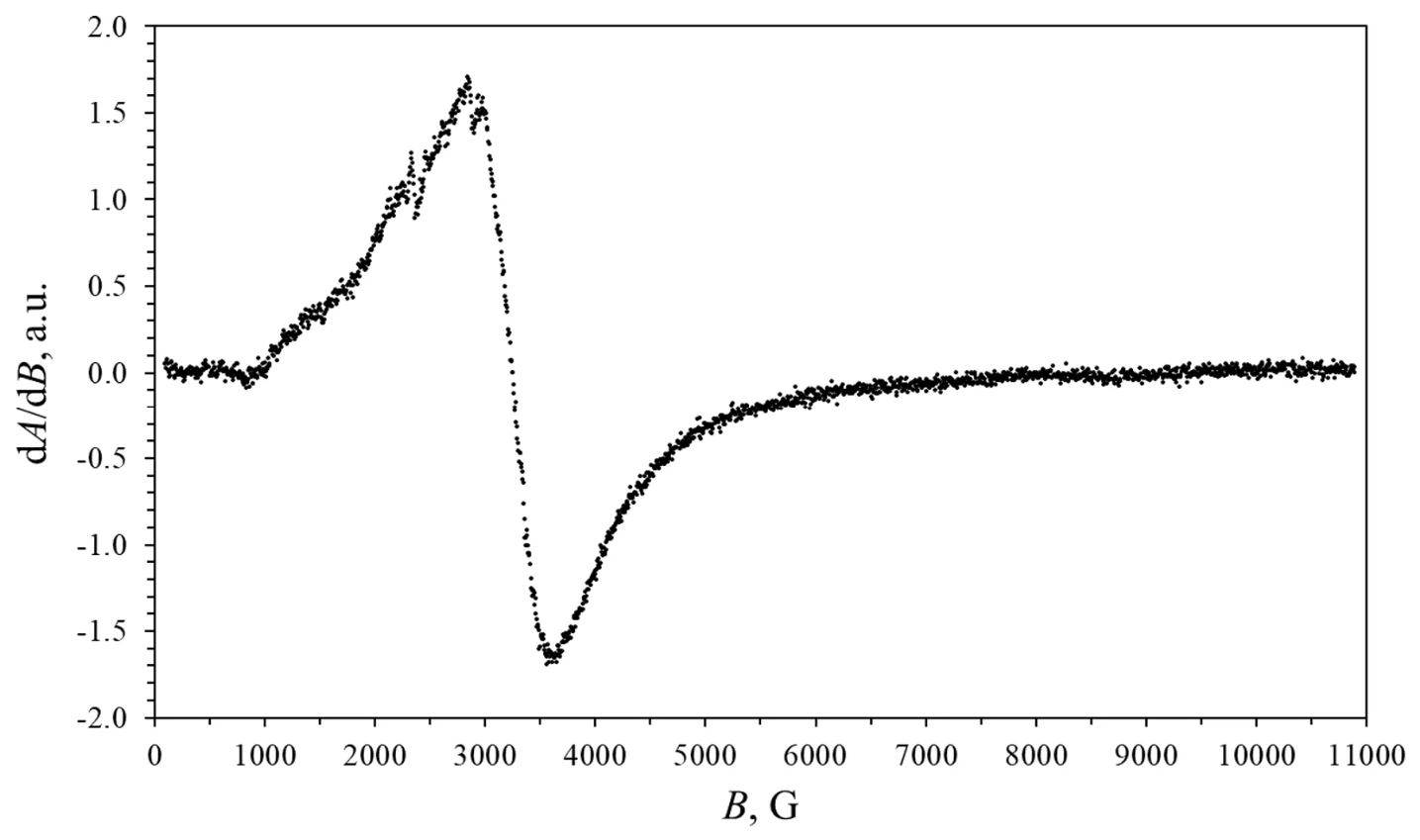

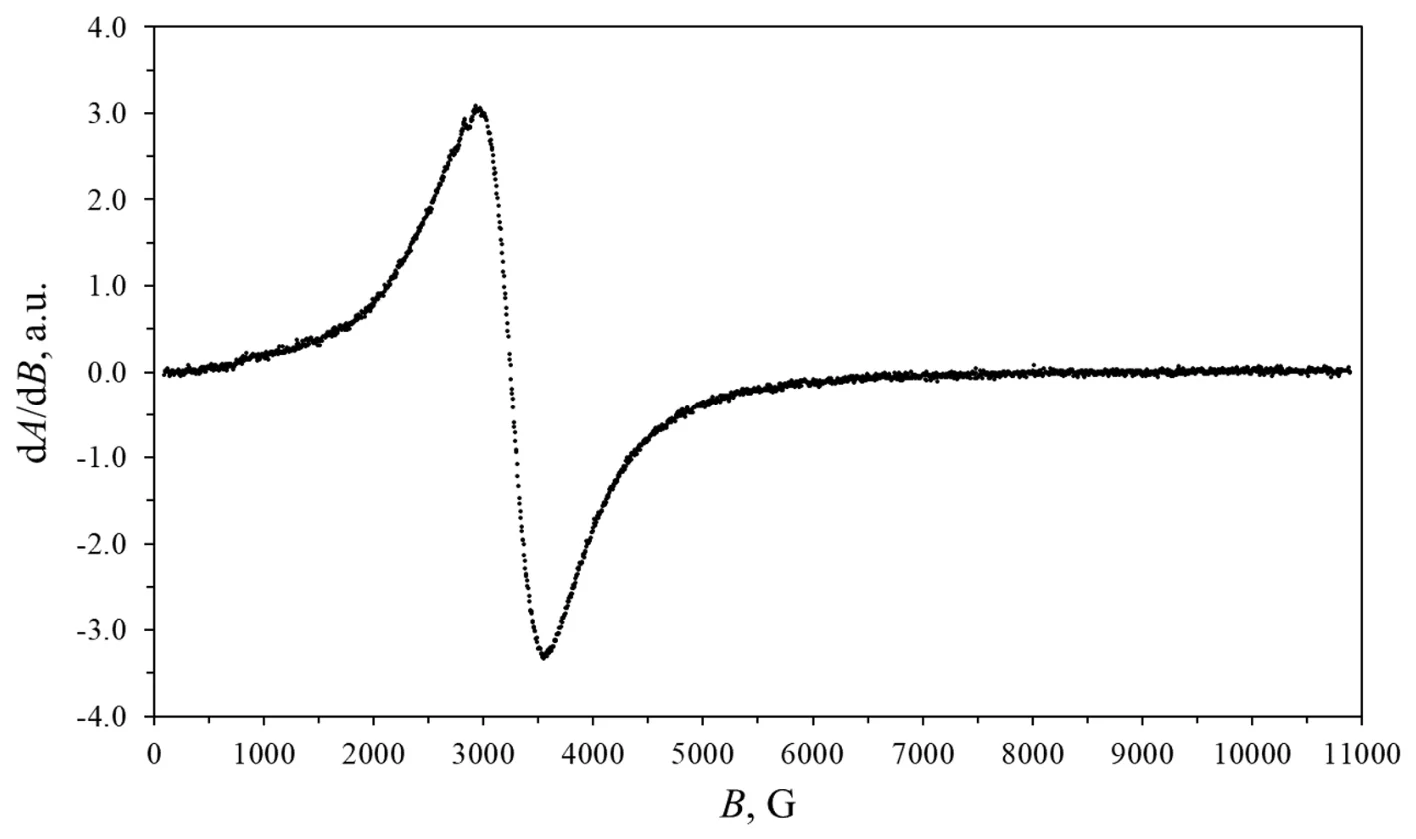
EPR spectra (g ≈ 2.0) of the 30 min Fenton reaction mixture: (**top**) without added (L)-malate; (**bottom**) with added (L)-malate, recorded under matched acquisition conditions; (field axes rescaled to f_0_ = 9.28 GHz and baseline-corrected. The malate-containing sample shows a ≈1.8-fold higher signal amplitude, consistent with a malate-dependent effect on paramagnetic Fe(III) speciation (Section 2.1.7).

**Figure 5:**
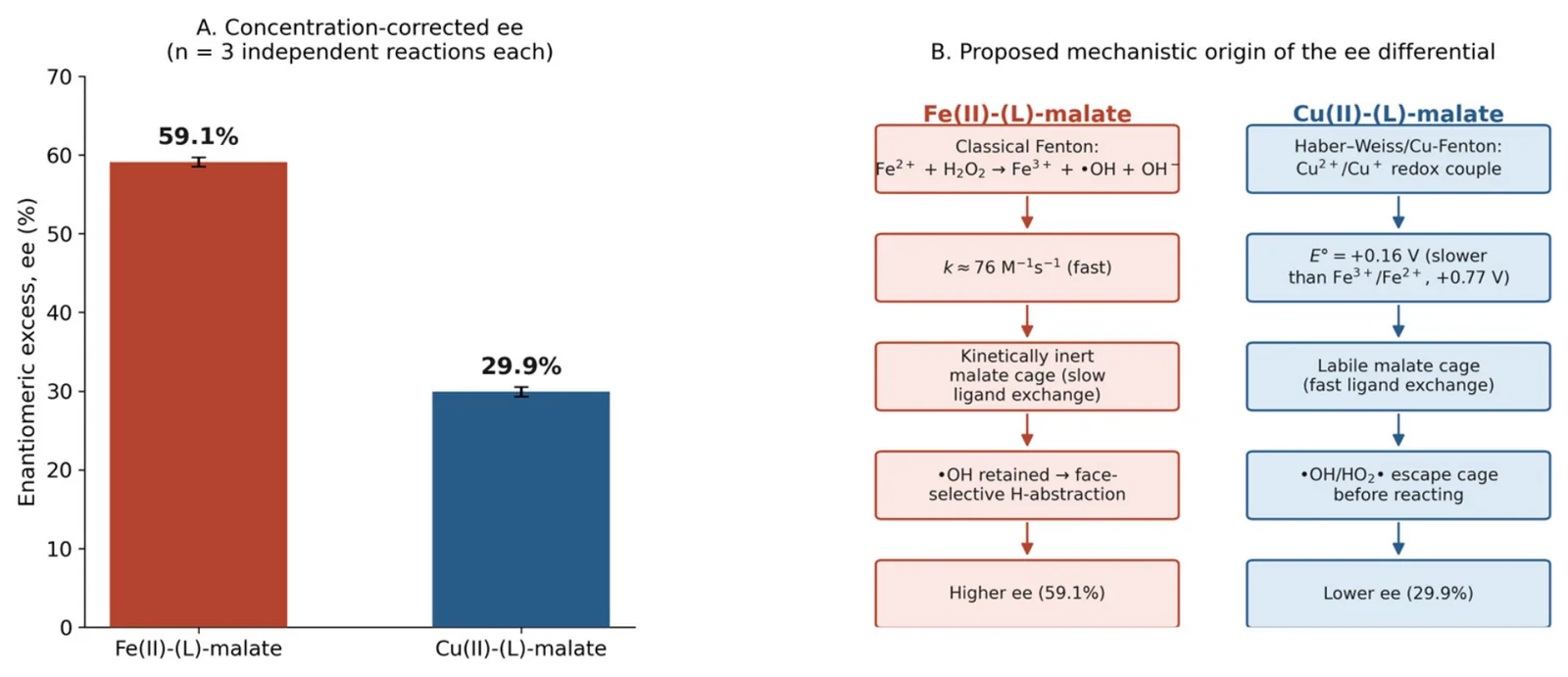
(**A**) Concentration-corrected enantiomeric excess (mean ± SD, *n* = 3) for Fe(II)- and Cu(II)-(L)-malate. (**B**) A proposed, not experimentally established, mechanistic model rationalising the Fe/Cu differential in terms of the relative kinetic inertness of the Fe(II)- versus Cu(II)-malate coordination sphere; presented as a working hypothesis for future testing (Section 3.3).

**Table 1:** (**a**) Optical rotation and apparent enantiomeric excess for Fe(II)- and Cu(II)-(L)-malate scaffolds. (**b**) Benedict’s-test-derived glyceraldehyde concentration and concentration-corrected specific rotation and enantiomeric excess (mean ± SD, *n* = 3 independent reactions per catalyst). (**c**) Acid-activation and catalyst-free control experiments (mean ± SD where *n* = 3).

(**a**)					
**Catalyst**	**Amount (mg)**	**[α]^20^D obs (°)**	**ee (%)**	**Mechanism**	
Fe(II)-(L)-malate	188	+5.0	57.5	Classical Fenton	
Cu(II)-(L)-malate	180	+2.5	28.7	Haber–Weiss/Cu-Fenton	
No H_2_SO_4_ (Fe, control)	188	0.00	0	No measurable activity	
(**b**)					
**Catalyst**	**α_obs_ (°), Raw (*n* = 3)**	**Benedict Consumption (mL, *n* = 3)**	**GLA (g/100 mL)**	**[α]_true_ (°)**	**ee (%)**
Fe(II)-(L)-malate	+4.96 ± 0.01	5.12 ± 0.08	0.964 ± 0.015	+5.14 ± 0.08	59.1 ± 0.9
Cu(II)-(L)-malate	+2.48 ± 0.01	5.06 ± 0.07	0.953 ± 0.013	+2.60 ± 0.04	29.9 ± 0.4
(**c**)					
**Control**	**α_obs_ (°), *n* = 3**	**Benedict Consumption (mL)**	**[α]_true_ (°)**	**ee (%)**	
Fe(II)-(L)-malate, no H_2_SO_4_	0.00 ± 0.00	0.00 ± 0.00	0.00	0.0	
Cu(II)-(L)-malate, no H_2_SO_4_	0.00 ± 0.00	0.00 ± 0.00	0.00	0.0	
Catalyst-free, Fe series (no metal, no malate) + H_2_SO_4_	0.00	0.00	0.00	0.0	
Catalyst-free, Cu series (no metal, no malate) + H_2_SO_4_	0.00	0.00	0.00	0.0	

**Table 2:** Effect of catalyst loading on observed optical rotation.

Catalyst	Loading	Mass (mg)	[α]^20^D obs (°)	Conversion (%)	Deviation from Standard
Fe(II)-(L)-malate	½ dose	94	+4.8	12.4	–4%
Fe(II)-(L)-malate	standard	188	+5.0	13.6	0%
Fe(II)-(L)-malate	2× dose	376	+5.2	17.8	+4%
Cu(II)-(L)-malate	½ dose	90	+2.3	11.7	–8%
Cu(II)-(L)-malate	standard	180	+2.5	13.5	0%
Cu(II)-(L)-malate	2× dose	360	+2.4	14.4	–4%

**Table 3:** Comparison of concentration-corrected enantiomeric excess (ee) obtained with pre-formed, isolated metal–malate salts (Table 1b, Section 2.1.2) versus the in situ FeSO_4_/CuSO_4_ + free (L)-malate control (this section), each with glyceraldehyde concentration determined independently by Benedict’s titration (*n* = 3 per condition).

Catalyst System	ee, Pre-Formed Salt (%)	ee, In Situ Control (%)	Δee
Fe	59.1 ± 0.9	58.7 ± 0.9	−0.4 (within error)
Cu	29.9 ± 0.4	28.5 ± 0.8	−1.4 (within error)

**Table 4:** Independent enantiomeric excess (ee) determined by triplicate (*n* = 3) preparative chiral column (Chiralpak IA) directly on the actual 30 min reaction aliquots (mean isomer masses, per-run SD = ±0.01 g; ee given as mean ± per-run SD propagated from this mass SD, the same statistic as in Table 1b), compared with the concentration-corrected ee from polarimetry (Table 1b). The ee values derive from weighed isolated fractions, not from analytical peak-area integration, and the method has not been formally validated.

Catalyst	D-(R) Isomer, Mean (g, *n* = 3)	L-(S) Isomer, Mean (g, *n* = 3)	Total Isolated (g)	ee, Chiral Column (%, *n* = 3)	ee, Polarimetry (%, Table 1b)
Fe(II)-(L)-malate	0.52 ± 0.01	0.13 ± 0.01	0.65	60.0 ± 2.5	59.1 ± 0.9
Cu(II)-(L)-malate	0.42 ± 0.01	0.23 ± 0.01	0.65	29.2 ± 2.3	29.9 ± 0.4

## Data Availability

Raw polarimetry data and the Python 3.9.6 figure-generation scripts supporting this article are available from the corresponding author upon reasonable request.
